# Acetylcholine as a shield: enhancing growth and salt tolerance in pepper through ionic homeostasis, hormonal regulation, antioxidant defence and gene expression

**DOI:** 10.1007/s00709-026-02223-9

**Published:** 2026-05-28

**Authors:** Esra Yaprak, Sumeyra Ucar, Oguzhan Araz, Ertan Yildirim, Melek Ekinci, Murat Aydin, Esma Yigider, Metin Turan, S. M. Zubair AL-Meraj, Totan Kumar Ghosh

**Affiliations:** 1 Department of Molecular Biology and Genetics, Erzurum Technical University, Erzurum, Turkey; 2 Department of Horticulture, Faculty of Agriculture, Atatürk University, Erzurum, Turkey; 3 Department of Agricultural Biotechnology, Faculty of Agriculture, Atatürk University, Erzurum, Turkey; 4 Department of Agricultural Trade and Management, Yeditepe University, Istanbul, Turkey; 5 Department of Crop Botany, Gazipur Agricultural University, Gazipur, Bangladesh

**Keywords:** Acetylcholine, Salinity, Antioxidant, Phytohormones, Nutrients, Transcripts

## Abstract

Acetylcholine (ACh), a potential neurotransmitter, shows growth and stress responses in plants. Despite, the underlying mechanisms of how ACh mitigates salt toxicity in pepper plants remains to be fully elucidated. Here, pepper plants pre-treated with different concentrations of ACh (0, 50 and 100 µM) were subjected to salinity stress (150 mM NaCl) to investigate their morpho-physiological, biochemical, and molecular alterations. Salinity stress significantly arrested plant phenotypes, reduced leaf relative water content (LRWC), and altered essential nutrient elements and phytohormones. However, it led to tremendous acceleration of proline, sucrose, hydrogen peroxide (H_2_O_2_), malondialdehyde (MDA), electrolyte leakage (EL), and enzymatic antioxidants such as superoxide dismutase (SOD), catalase (CAT), peroxidase (POD), and glutathione reductase (GR) as compared to the non-stress control plant. ACh greatly improved the plant’s phenotypes in non-stress control plants and significantly repaired the phenotype of salinity-affected plants. ACh improved LRWC and plant nutrient status by restricting Na^+^ and Cl^−^. However, it stimulated synthesis of phytohormones (except ABA), and bolstered the functions of SOD, CAT, POD, and GR under salinity stress. While salinity stress upregulated *CaHKT1;1* and *CaHKT1;2*, ACh treatment downregulated them. Compared to control, salinity and ACh exposure stimulated the expression of genes governing Photosystem II (PSII) and antioxidant activity, highlighting a coordinated genetic defence mechanism of plant while mitigating salinity stress. However, significant induction of *CaPsbD* by ACh under salinity stress suggests the protective role of this phytochemical in PS II recovery. Furthermore, multivariate analysis demonstrated that application of ACh (100 µM) improved most of the physiological and biochemical markers, effectively restoring plant growth despite salinity stress. The findings imply a crucial role for ACh in growth and mitigation of salt toxicity in the pepper plant.

**Supplementary Information:**

The online version contains supplementary material available at 10.1007/s00709-026-02223-9.

## Introduction

As sessile organisms, plants are frequently exposed to a variety of abiotic stresses, including salinity. Of the abiotic stressors, soil salinity is a major global concern affecting about 20% of usable agricultural land and causing tremendous negative consequences in agricultural productivity and food security (Ei sabagh et al. 2021; Singh 2022). The scenario has been exacerbated due to gradual changes in the world’s climate and improper agricultural practices (Khamidov et al. 2022). Soil salinity is a major factor that diminishes plant productivity in most arid and semi-arid areas of the world. It has tremendous negative consequences on plant morpho-physiological, biochemical, and entire growth and developmental processes. Salinity, especially high levels of NaCl, increases osmotic stress in plants and reduces their capacity to take up water from the soil, leading to injury and even death (Mohammed et al. 2022). Salinity restricts important physiological and biochemical processes in plants like transpiration, photosynthesis, respiration, cell division, membrane functioning, ionic balance and gene expression process (Saad-Allah et al. 2022; Hamouda et al. 2023; Abbas et al. 2024). Salinity-mediated stunting of plant growth has been widely investigated across various crop species (Kaushal and Wani 2016; Ucar et al. 2026). The most stunning effect of the salinity stress is to develop higher Na^+^/K^+^, thereby inhibiting the availability of other essential nutrient elements. However, it triggers the generation of reactive oxygen species (ROS), particularly hydrogen peroxide (H_2_O_2_), the lipid peroxidation product malondialdehyde (MDA), and electrolyte leakage in plants (Akter et al. 2023; Zhang et al. 2023). To counter the salinity problem, plants employ several inherent strategies, such as increased accumulation of compatible solutes, elevated synthesis of phytohormones, enhanced antioxidant systems, and increased expression of stress-responsive genes (Ghosh et al. 2021; Akter et al. 2023; Qiao et al. 2024). Despite those mechanisms, it is insufficient for plants to acclimate and survive under extreme salinity stress. Several agronomic practices, including improving existing soil and developing salt-tolerant varieties, are limited in scope due to the time, labor, effort, and substantial investment required. However, the use of phytochemicals and microorganisms has been a popular strategy over the past couple of years to enhance plants’ capacity to withstand abiotic stresses, including salinity. For instance, the application of salicylic acid, polyamines, humic acid, fulvic acid, mycorrhizae, and bacteria has been investigated previously to confer salinity tolerance in plants (Kaushal and Wani 2016; Zhao et al. 2021; Shen et al. 2024; Subhi et al. 2025). Despite this, the implications of more convenient and eco-friendly chemical approaches are required to boost productivity under salt stress.

The concept of neurotransmitter comprised with melatonin, serotonin, dopamine, gamma-aminobutyric acid (GABA) and Acetylcholine (ACh) which was initially discovered in fungi, later implicated in mammals as a signaling molecules—which has become popular in plant system over the time for attaining regulatory roles in plant growth, development and stress acclimation process (Bouche and Fromm 2004; Qin et al. 2020, 2021; Ghosh et al. 2025; Ucar et al. 2026). Of that neurotransmitter, ACh, the most popular one, predominantly synthesized in animal by canonical neuronal signaling pathway (Roychoudhury 2020) but can also be synthesized in the non-neuronal cells of a wide range of organisms including bacteria, algae and plants (Su et al. 2020b). Along with other neurotransmitters, ACh shows diverse functions in regulating plant growth, development, and stress responses (Su et al. 2020b). It showed tremendous impacts on photosynthesis, seed germination, shoot and root development, osmoregulation, and antioxidant defence mechanisms against many abiotic stresses, including fluctuating weather, heavy metal, and drought stress (Qin et al. 2021). While addressing abiotic stresses, ACh was demonstrated to be linked with phytohormones and stress-responsive transcripts (Di Sansebastiano et al. 2014; Qin et al. 2021; Shen et al. 2024). Nevertheless, further clarification is required to elucidate the mechanistic insights through phytohormone profiling and transcriptomic studies. In contrast to other abiotic stresses, the implication of this signaling molecule is limited to salt-stressed plants. For instance, ACh was reported to enhance salt tolerance in wheat and tobacco by improving physiological makeup, particularly by destabilizing ROS via the antioxidant-mediated defence system (Qin et al. 2019; Shen et al. 2024). Despite, the deeper understanding of how ACh alleviates salinity stress by modulating plants’ nutrients, phytohormones, and the expression of Na^+^ and K^+^ transporters, PS-II, and enzymatic antioxidant-related transcripts remains to be clarified. However, implication of ACh has yet to be comprehensively investigated in a high value spice crop.

Pepper (*Capsicum annuum* L.), belonging to the genus Capsicum of the Solanaceae family, has been widely cultivated in both temperate and tropical climates around the world (Altunlu 2020). It has been a significant agricultural product having its crucial values for nutritional, economic and commercial purposes. The fruit of the crop is rich in vitamin C and antioxidants having tremendous positive impacts on human health (Sariyer and Çiftci 2022). Although having those potentials, the concerning issue is that the crop shows a moderate level of sensitivity to salinity stress (Altunlu 2020; López-Serrano et al. 2021). The growth and development of the crop have been greatly affected by salinity stress induced by elevated NaCl (Altunlu 2020; Subhi et al. 2025). Thus, the application of potential phytochemicals like ACh may enhance salinity tolerance in pepper plants, although it has yet to be demonstrated. Therefore, this study aimed to pre-treating pepper plants with ACh prior to salinity stress (150 mM NaCl) to elucidate the role of this signaling molecule in stress recovery. By evaluating water and nutritional status, ROS dynamics, antioxidant systems, phytohormone profiling, and analyzing stress- responsive transcripts (ion transporters, PS-II, and antioxidant genes)—this effort offers a comprehensive perspective that distinguishes it from the previous studies. The study would elucidate the mechanistic basis of ACh- mediated salt tolerance in plants and generate novel strategies that could support the sustainable growth of pepper plants worldwide.

## Materials and methods

### Plant materials and imposition of NaCl and ACh treatments

The study was conducted in the Department of Horticulture at Atatürk University. Seeds of Pepper (*Capsicum annuum* L. cv. Yalova) were germinated in small pots filled with a peat-perlite mixture (2:1, v/v) under greenhouse conditions. The greenhouse’s temperature and humidity were adjusted using a sensor, which maintained an average of 24 (± 2) °C during the day and 18 (± 2) °C at night, and 50% humidity. When the seedlings reached the three-true-leaf stage, they were transferred to pots (12.5 cm diameter, 12.5 cm depth) containing a soil: sand: peat mixture (2:1:1). The pots were randomly placed in the greenhouse by maintaining a completely randomized design (CRD) where each pot contained one plant, and three pots were considered as a single replication. The experiment was conducted with three replicates. After 5 days of plants’ acclimation in the pot, acetylcholine (ACh) was applied as a foliar spray at different concentrations (0, 50, and 100 µM) (Qin et al. 2019; Arikan-Abdulveli 2025), and a total of three applications were repeated at 5-day intervals. A 0.1% (v/v) Tween-20 solution was included in ACh solution to facilitate absorption by the leaves. After 24 h of last application of ACh, irrigation water supplemented with 150 mM NaCl (Yildirim et al. 2022) was applied to the plants to induce salinity stress. Plants without ACh and NaCl constituted the non-stress control group. The treatment combinations followed in this investigation were: T1: control without NaCl and ACh (C), T2: 50 µM ACh under control condition (C + 50 µM ACh), T3: 100 µM ACh under control condition (C + 100 µM ACh), T4: 150 mM NaCl, T5: 150 mM NaCl + 50 µM ACh, and T6: 150 mM NaCl + 100 µM ACh.

### Phenotypic data collection

The morphological data on plant height (PH), shoot fresh weight (SFW), shoot dry weight (SDW), root fresh weight (RFW), and root dry weight (RDW) were recorded after 30 days of treatment imposition. While taking root data, those were carefully washed and deliberately handled to avoid root damage and all sort of debris. The RFW was measured after drying the surface water. Then, both roots and shoots were oven-dried at 70 °C for two days to measure the dry weight of the plant’s samples. The data were collected by maintaining three replications. The collected leaf samples were either used directly for physiological assays or frozen in liquid nitrogen and stored at -80 °C for biochemical and molecular analyses.

### Measurements of leaf relative water content (LRWC) and photosynthetic pigments

The percentage (%) of LRWC was calculated by following the procedure of Shivakrishna et al. (2018). In a brief, fresh weight of the collected leaf samples was recorded from each treatment and allowed to petri dishes containing distilled water for 24 h at room temperature in the dark. The turgid weight of the leaf was recorded after blotting the leaf’s surface water. Dry weight of the leaf sample was determined after oven drying at 70 °C for 24 h. The LRWC (%) was calculated using the formula: RWC (%) = [(FW ˗ DW) / (TW ˗ DW)] × 100. Where, FW = Fresh weight, DW = Dry weight, and TW = Turgid weight of the leaf sample. The photosynthetic pigments, such as chlorophylls and carotenoids were determined using the method followed by Porra et al. (1989).

### Determination of proline, soluble sugar, hydrogen peroxide (H_2_O_2_), malondialdehyde (MDA) and enzymatic antioxidants

Proline content in the fresh pepper’s leaf was determined by following methodology of Bates et al. (1973) and expressed as µmolg^− 1^ FW. Sucrose content was determined from the dry leaf samples by following the methodology of Yuce et al. (2024), and calculated as % dry weight (DW). H_2_O_2_ and MDA content was determined according to the methods followed by Ghosh et al. (2021). The superoxide dismutase (SOD, EC: 1.15.1.1) activity was measured as followed by Spitz and Oberley (1989). The catalase (CAT, EC: 1.11.1.6) activity was measured at 240 nm using an extinction coefficient of 39.4 M^− 1^ cm^− 1^ by following the methods of Noctor et al. (2016). The guaiacol peroxidase (POD, EC: 1.11.1.7) activity was quantified at 470 nm using an extinction coefficient of 26.6 mM^− 1^ cm^− 1^ as followed by Castillo et al. (1984). The activities of glutathione reductase (GR, EC: 1.6.4.2), were measured at 340 nm using extinction coefficients of 6.2 mM^− 1^ cm^− 1^ and by following the methods of Noctor et al. (2016). The reaction kinetics for each enzymatic assay was followed by 1 min reaction.

### Phytohormones profiling

For the phytohormone profiling, extraction and purification of the dry leaf samples were done according to the methodology followed by Koshita et al. (1999). Phytohormones like indole-3-acetic acid (IAA), gibberellic acid (GA), cytokinins; kinetin (Kin) and zeatin (Zea), jasmonic acid (JA), salicylic acid (SA) and abscisic acid (ABA) were quantified by High-Performance Liquid Chromatography (HPLC) with a Zorbax Eclipse-AAA C-18 column (Agilent 1200 HPLC). Then, occurrence of specific phytohormone was detected by following the methodology of Ekinci et al. (2023). The presence of individual phytohormone was calculated as ng g^-1^ DW.

### Determination of essential nutrient elements

The presence of essential macro- and micro-elements were determined from the dry leaf samples of pepper plant. The content of Nitrogen (N) was determined by Kjeldahl method. Analysis of the occurrence of other nutrients elements like phosphorus (P), potassium (K), calcium (Ca), magnesium (Mg), sulfur (S), manganese (Mn), iron (Fe), zinc (Zn), boron (B), chlorine (Cl), sodium (Na) and copper (Cu) in the leaf samples was measured by following the method of Mertens et al. (2005). The macronutrients (N, P, K, Ca, Mg, and S) were calculated as % DW and micronutrients (Fe, Mn, Zn, B, Cl, and Cu) and Na were recorded as mg kg^− 1^ DW.

### Analysis of abiotic stress-responsive transcripts

RNA extraction kit (Sigma-Aldrich, St. Louis, MO, USA) was used to extract total RNA from the frozen leaf samples. The quality of RNA was validated using a spectrophotometer (Thermo Multiskan GO 1510, Finland) and allowed to 1% agarose gel electrophoresis. The first-stand cDNA was synthesized from total RNA (1 µg) using cDNA synthesis kit (Thermo Scientific) for quantitative PCR (qPCR) assay. The primer pairs for the target stress-related genes (*CaHKT1;1*, *CaHKT1;2*, *CaPsbA*, *CaPsbB*, *CaPsbC*, *CaPsbD*, *CaACT*, *CaCu/ZnSOD*, *CaPOD*, *CaAPX*, *CaGR* and *CaCAT*) were constructed based on Phytozome (https://phytozome.jgi.doe.gov/) online tool (Supp. Table 1). The reaction mixture (20 µL solution containing 2 µL diluted cDNA sample, 10 µL SYBR green master mix (Qiagen), 0.1 µL forward and reverse primers (100 pmol) and RNase-free H_2_O) was used to perform qPCR assay. The reaction condition (10 min at 95 °C, 40 cycles of 15 s at 95 °C, and 60 s at 60 °C) was followed as maintained by Ekinci et al. (2023). *β-actin* reference gene was used to normalize the data and the expression of the transcripts was estimated using 2^−ΔΔCt^ (2-delta delta Ct) method (Livak and Schmittgen 2001).

### Statistical analysis

The experiment was followed by completely randomized design (CRD) with three replications. One-way ANOVA was used to analyse the data using R program (Rv.4.2.2 for Windows (http://CRAN.R-project.org/). Tukey’s Honestly Significant Difference (HSD) was used for mean comparison analysis. The library pheatmap was used to generate a heatmap and hierarchical clusters (Kolde 2019) using R-studio. The PCA was performed using ggplot2, factoextra, and FactoMineR (Lê et al. 2008; Wickham 2016).

## Results

### ACh improves growth responses in pepper plant

**Fig. 1:**
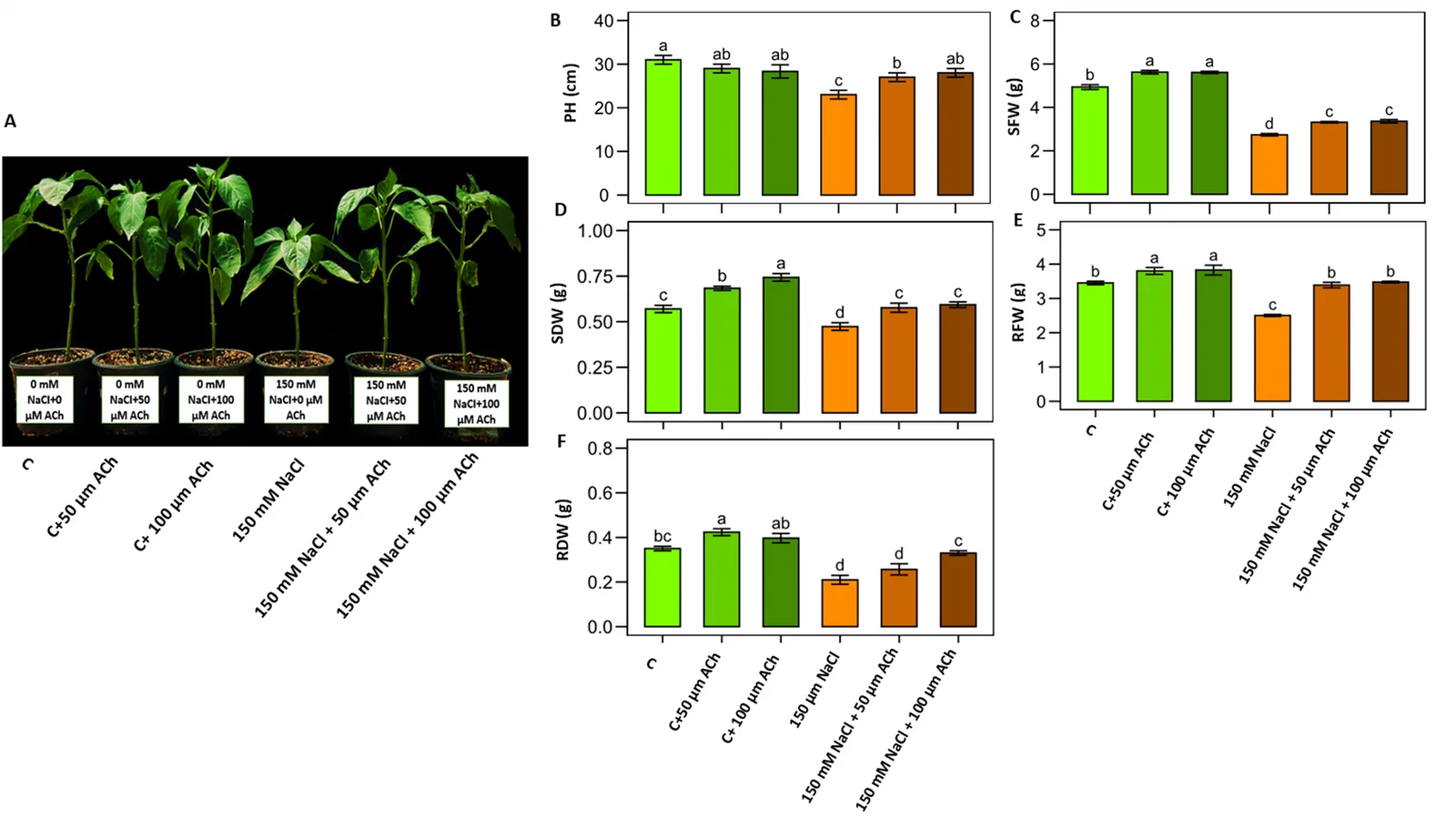
Effects of acetylcholine (ACh) on the phenotypic appearances and growth responses of pepper seedlings. Phenotypic appearance (**A**), plant heigh; PH (**B**), shoot fresh weight; SFW (**C**), shoot dry weight; SDW (**D**), root fresh weight; RFW (**E**), and root dry weight; RDW (**F**) of pepper plant subjected to non-stress control (**C**), salinity stress (150 mM NaCl), and different concentrations of ACh (50 and 100 µM) under non-stress control and salinity-stressed conditions. Vertical bars represent +/− standard error (SE) values. Different letter(s) on the bar indicate a significant difference at *p* < 0.001

To assess the effects of ACh on plant growth and development under non-stress control and salt-stressed conditions, we recorded the data of plant height (PH), shoot fresh weight (SFW), shoot dry weight (SDW), root fresh weight (RFW), and root dry weight (RDW). The phenotype of pepper plant was greatly recovered upon exogenous application of ACh (Fig. 1A). The results demonstrated that salt-stress (150 mM NaCl) significantly reduced those plant attributes by 25.8%, 44.5%, 16.9%, 27.4% and 40.0% respectively, as compared to control plants (Fig. 1B and F). Exogenous ACh improved plant parameters under both non-stress control and salt-stressed conditions. ACh (50 µM) greatly enhanced the above-mentioned parameters by 17.4%, 21.0%, 21.8%, 35.4%, and 22.2% respectively when compared to only salt-treated plants (Fig. 1B and F). The plant phenotypes were found to be upgraded further by increasing concentration of ACh. The results clearly demonstrated the conspicuous role of ACh in improving growth of pepper plant under both optimal and salt-stressed conditions.

### ACh stabilizes photosynthetic pigments in pepper under salinity stress

**Fig. 2:**
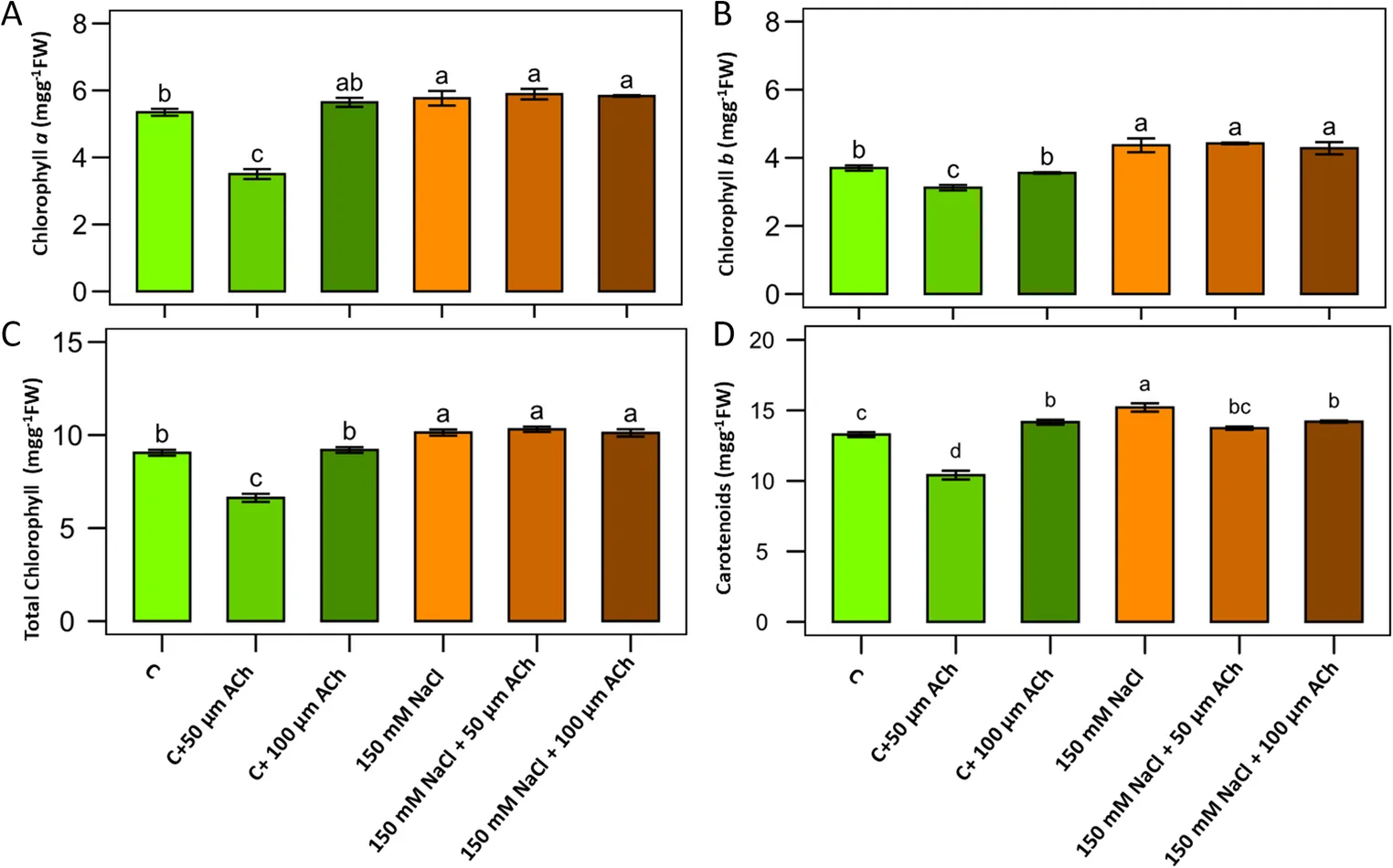
Acetylcholine (ACh)-mediated modulation of photosynthetic pigments. Cholorophyll *a* (**A**), chlorophyll *b* (**B**), total chlorophyll (**C**), and carotenoids content (**D**) of pepper plant subjected to non-stress control (**C**), salinity stress (150 mM NaCl), and different concentrations of ACh (50 and 100 µM) under non-stress control and salinity-stressed conditions. Vertical bars represent +/− standard error (SE) values. Different letter(s) on the bar indicate a significant difference at *p* < 0.001

As compared to non-stress control plants, salinity stress significantly enhanced levels of chlorophyll a (Chl *a*), chlorophyll b (Chl *b*) and total chlorophyll (TChl) by 7.8%, 18.0% and 12.0% respectively (Fig. 2A and C). Although exogenous ACh maintained little increase of both *Chl a* and *Chl b* but significant increment of TChl content (12.6%) reflecting that ACh could stabilize chlorophyll pigments in leaf under salt stress (Fig. 2A and C). Along with that, carotenoids content was significantly improved by salt stress (14.5% compared to control plant) which was further declined (by 9.6%) upon application of 50 µM ACh (Fig. 2D). However, ACh (100 µM) led to increase carotenoids by 6.6% under non-stress control condition, suggesting differential responses of carotenoids to ACh depending on growth conditions.

### ACh improves nutrient status in plant

**Fig. 3:**
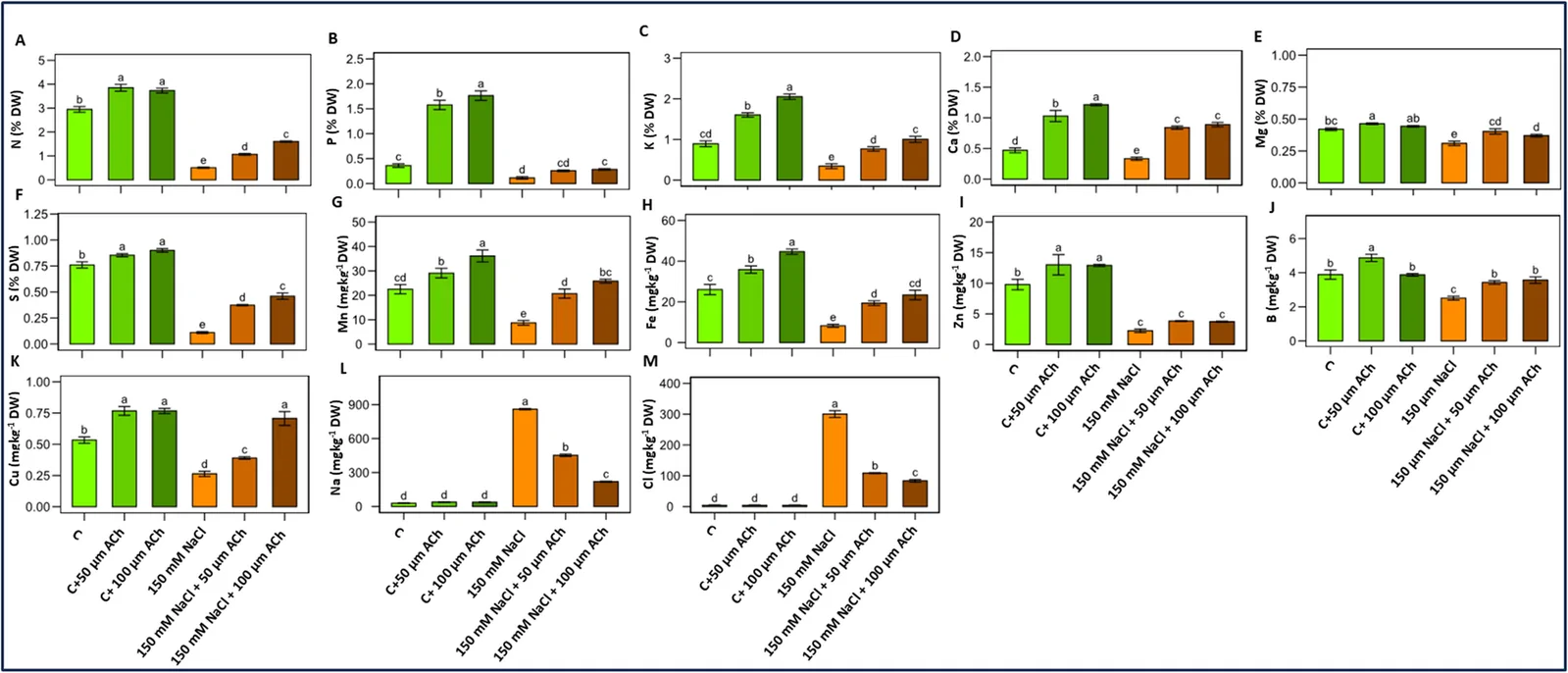
Regulation of acetylcholine (ACh) on the availability of nutrient elements in pepper leaf. Percentage (%) of nitrogen; N (**A**), phosphorus; P (**B**), potassium; K (**C**), calcium; Ca (**D**), magnesium; Mg (**E**), sulfur; S (**F**), manganese; Mn (**G**), iron; Fe (**H**), zinc; Zn (**I**), boron; B (**J**), cupper; Cu (**K**), sodium; Na (**L**), and chloride; Cl (**M**) in the leaf of pepper plant subjected to non-stress control (**C**), salinity stress (150 mM NaCl), and different concentrations of ACh (50 and 100 µM) under non-stress control and salinity-stressed conditions. Vertical bars represent +/− standard error (SE) values. Different letter(s) on the bar indicate a significant difference at *p* < 0.001

To evaluate the impacts of exogenous ACh on nutrient elements under salt-stress, we estimated macro and micro-elements in pepper’s leaf. Salt stress dramatically declined the essential nutrient elements in pepper leaf. It caused significant reduction of N, P, K, Ca, Mg, S, Mn, Fe, Zn, B, and Cu by 82.8%, 68.5%, 61.5%, 29.1%, 26.2%, 85.5%, 61.2%, 68.3%, 77.0%, 35.5%, and 50.6% respectively, as compared to control (Fig. 3A and K). In contrast, salinity stress led to mount Na and Cl by many folds (Fig. 3L and M). Exogenous ACh showed significant increment of essential nutrient elements in plant under control condition reflecting its crucial role for the growth and development of pepper plant. Tremendous recovery of those elements by 215.8%, 150.0%, 192.2%, 167.0%, 19.4%, 318.2%, 195.9%, 183.1%, 66.7%, 42.2%, and 168.4% respectively was recorded when plants were treated with 100 µM ACh under salt-stress (Fig. 3A and K). However, significant dropped down of the accumulation of Na (74.5%) and Cl (72.1%) (Fig. 3L and M) by 100 µM ACh reflecting the potential of this phytochemical in maintaining nutrient homeostasis and safeguarding essential elements under salt stress.

### ACh alters leaf relative water content and accumulation of osmolytes

**Fig. 4:**
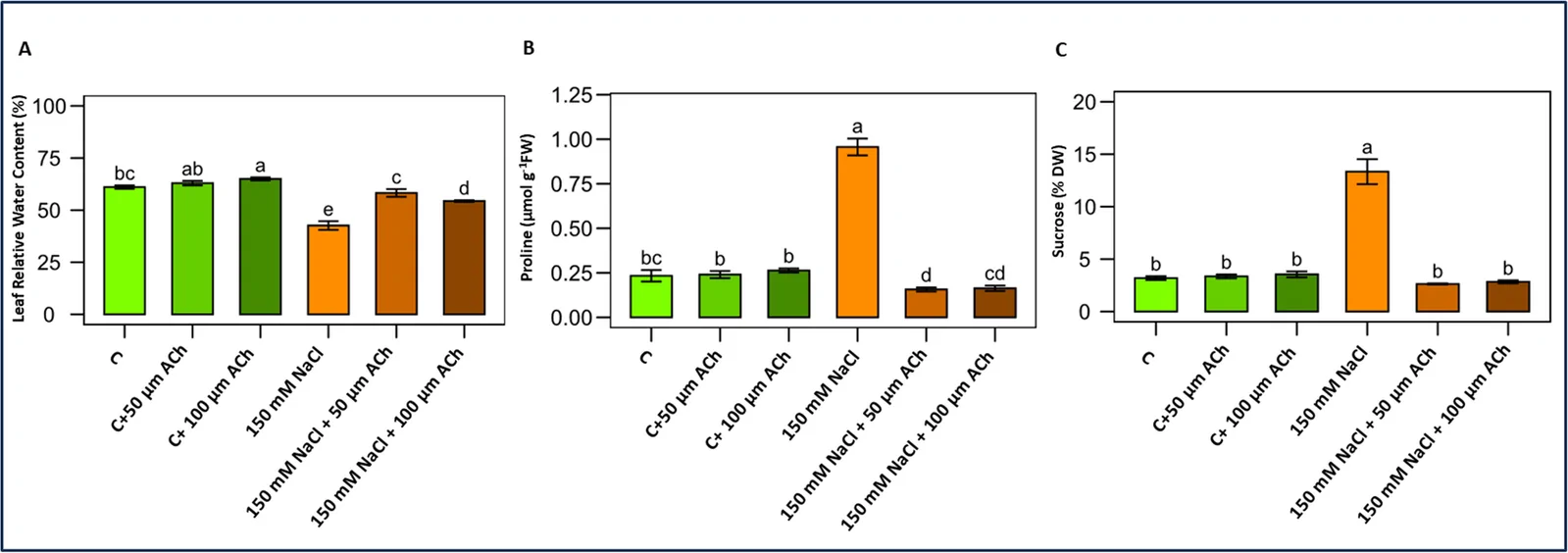
Effects of acetylcholine (ACh) on the leaf relative water content (%) (**A**), and accumulation of osmolytes; proline (**B**) and sucrose (**C**) in pepper plants subjected to non-stress control (**C**), salinity stress (150 mM NaCl), and different concentrations of ACh (50 and 100 µM) under non-stress control and salinity-stressed conditions. Vertical bars represent +/− standard error (SE) values. Different letter(s) on the bar indicate a significant difference at *p* < 0.001

We measured leaf relative water content; LRWC (%) and osmolytes proline and soluble sugar sucrose in pepper leaves. Although LRWC (%) was more or less similar under optimal condition even after application of ACh, salt-stress greatly impacted on it (reduced by 30.2% compared to non-stress control plant) (Fig. 4A). However, exogenous ACh at both 50 and 100 µM significantly restored LRWC (%) in salt-affected plants (Fig. 4A). As compared to control plant, both proline and sucrose content was dramatically enhanced by 310.0% and 318.1% under salt-stress (Fig. 4B and C). However, significant reduction of that accumulation (83.6% and 80.3%) was recorded by 50 µM ACh.

### ACh mitigates oxidative damage in salt-stressed pepper plant

**Fig. 5:**
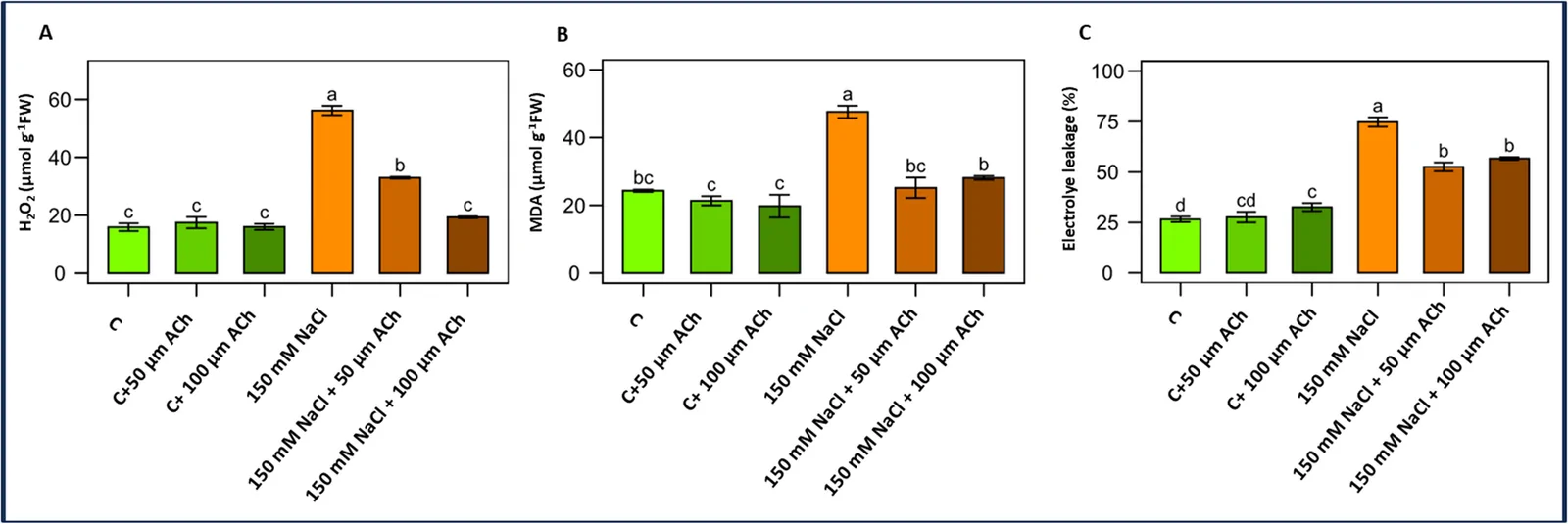
Reduction of salt-induced oxidative damage by acetylcholine (ACh). The content of hydrogen peroxide; H_2_O_2_ (**A**), lipid peroxidation product; malondialdehyde; MDA (**B**) and electrolyte leakage (EL) in pepper plants subjected to subjected to non-stress control (**C**), salinity stress (150 mM NaCl), and different concentrations of ACh (50 and 100 µM) under non-stress control and salinity-stressed conditions. Vertical bars represent +/− standard error (SE) values. Different letter(s) on the bar indicate a significant difference at *p* < 0.001

We measured incidence of ROS in terms of H_2_O_2_, and lipid peroxidation product; MDA and electrolyte leakage; EL (%) from the damaged tissue. Salt-stress caused significant induction of H_2_O_2,_ MDA and EL (%) by 253.5%, 95.6% and 181.5% as compared to non-stress control plant (Fig. 5). No significant differences of the accumulation of those were found in control plants and control plants treated with ACh (Fig. 5). In contrast, ACh showed prominent suppression of H_2_O_2_ (by 41.3%), MDA (by 47.0%) and % EL (by 29.7%) while plants were subjected to salt-stress (Fig. 5). Thus, the results suggesting that exogenous ACh could produce tremendous impacts in destabilizing ROS in pepper plant under salt-stress.

### ACh promotes enzymatic antioxidant in salt-stressed pepper plant

**Fig. 6:**
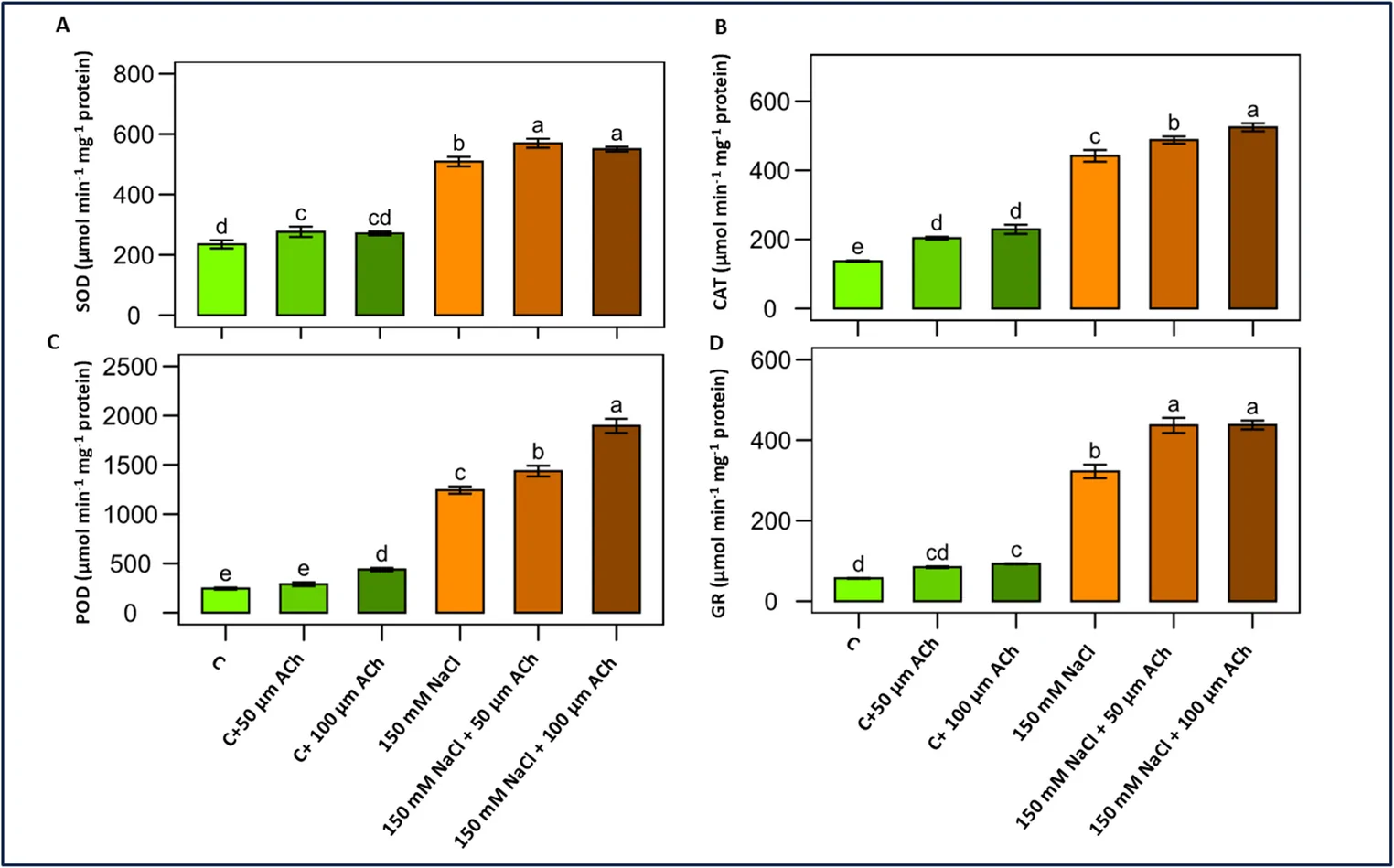
Acceleration of enzymatic antioxidants by acetylcholine (ACh) under salt stress. The activity of superoxide dismutase; SOD (**A**), catalase; CAT (**B**), peroxidase; POD (**C**) and glutathione reductase (GR) in pepper plants subjected to non-stress control (**C**), salinity stress (150 mM NaCl), and different concentrations of ACh (50 and 100 µM) under non-stress control and salinity-stressed conditions. Vertical bars represent +/− standard error (SE) values. Different letter(s) on the bar indicate a significant difference at *p* < 0.001

To see the functions of enzymatic antioxidants, we recorded the data of activities of SOD, CAT, POD and GR in the control and salt- affected plants. As compared to non-stress control, the activity of SOD, CAT, POD and GR was markedly enhanced by 222.5%, 406.9%, 116.8% and 465.8% respectively in pepper’s leaf treated with 150 mM NaCl (Fig. 6). The activity was further heightened by exogenous application of ACh as evidenced by the increment of those activities by 10.4%, 15.5%, 11.9% and 35.5% in 50 µM ACh treated plants under salinity stress (Fig. 6). Exogenous ACh produced little effects on the activities of enzymatic antioxidants under non-stress conditions. Thus, the findings indicated a crucial role of ACh in improving enzymatic antioxidant defence in pepper plant while subjected to salinity stress.

### ACh accelerates phytohormones biosynthesis in pepper plant

**Fig. 7:**
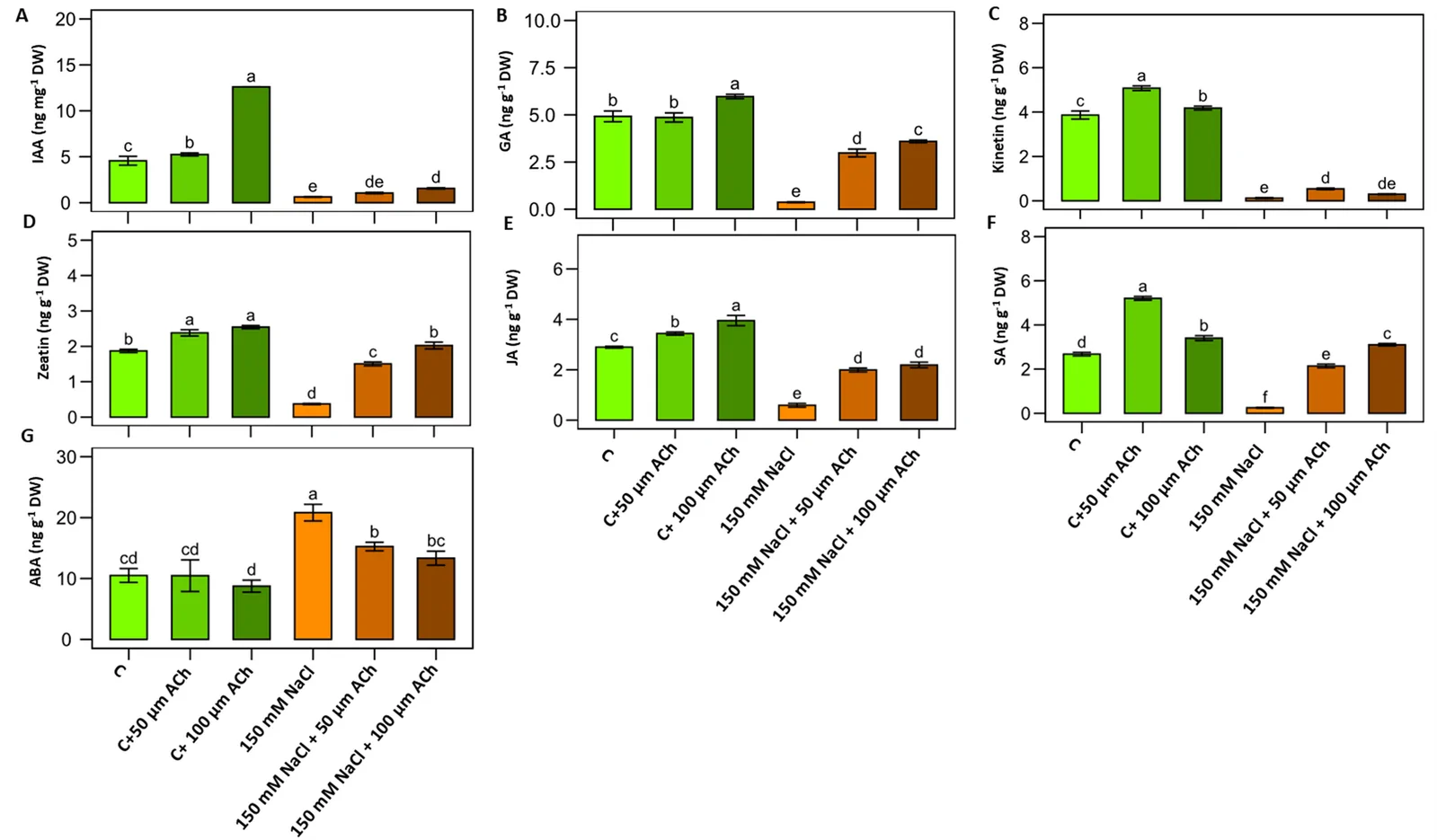
Effects of exogenous acetylcholine (ACh) on the biosynthesis of phytohormones. Indole-3-acetic acid; IAA (**A**), gibberellic acid; GA (**B**), kinetin (**C**), zeatin (**D**), jasmonic acid; JA (**E**), salicylic acid; SA (**F**) and abscisic acid; ABA (**G**), content of pepper plants subjected to non-stress control (**C**), salinity stress (150 mM NaCl), and different concentrations of ACh (50 and 100 µM) under non-stress control and salinity-stressed conditions. Vertical bars represent +/− standard error (SE) values. Different letter(s) on the bar indicate a significant difference at *p* < 0.001

Because phytohormones act as the crucial signalling components during abiotic stresses including salinity, we investigated phytohormones profiling of the treated and non-treated leaf extracts. Essential phytohormones like IAA, GA, Kin, Zea, JA and SA were sharply declined in the plants supposed to salinity stress and content of those phytohormones were decreased by 86.4%, 92.5%, 96.9%, 80.2%, 79.5% and 90.7% respectively compared to non-stress control plants (Fig. 7A and F). Contrary, the content of ABA was sharply induced by 98.4% under salinity stress (Fig. 7G). Exogenous ACh produced significant induction of those phytohormones under both non-stress control and salinity stressed conditions except ABA. ACh (50 µM) displayed significant increment of IAA, GA, Kin, Zea, JA and SA by 68.4%, 705.4%, 347.2%, 306.3%, 235.4% and 757.3% respectively under salinity stress (Fig. 7A and F). The effect of ACh was found to be antagonistic to ABA under salinity stress and content of which was reduced by 26.8% upon 50 µM ACh (Fig. 7G). The findings suggested the crucial regulation of phytohormone biosynthesis by ACh irrespective of growing conditions.

### ACh modulates gene expression related to ion transporters, PS II, and antioxidant enzymes

**Fig. 8:**
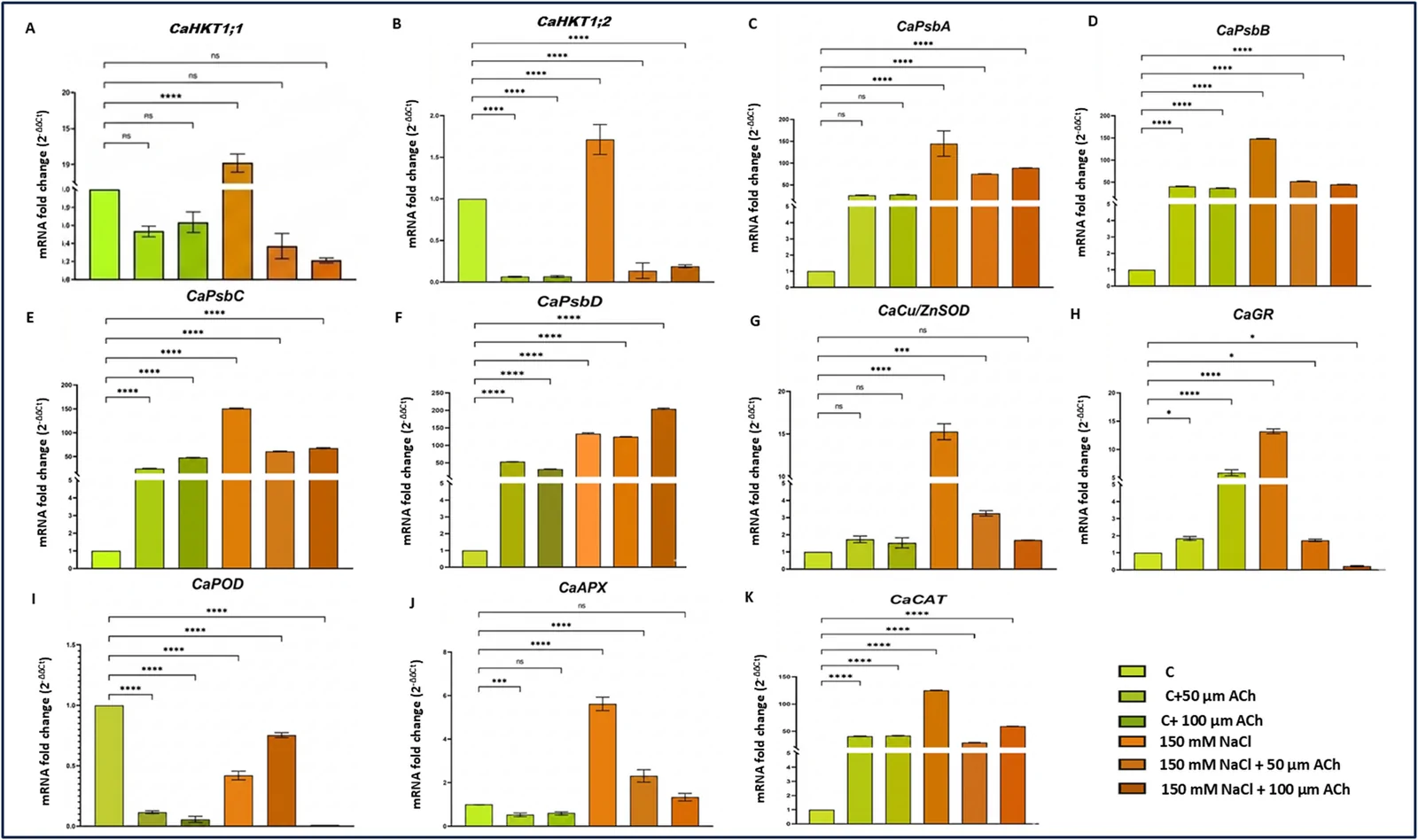
Modulation of expression of transcripts related to high affinity potassium transporter (HKT), photosystem II (PS II), and antioxidant enzymes in pepper leaf. Expression of *CaHKT2;1* (**A**), *CaHKT2;2* (**B**), *CaPsbA* (**C**), *CaPsbB* (**D**), *CaPsbC* (**E**), *CaPsbD* (**F**), *CaCu/ZnSOD* (**G**), *CaGR* (**H**), *CaPOD* (**I**), *CaAPX* (**J**) and *CaCAT* (**K**) in the leaf of pepper plant subjected to non-stress control (**C**), salinity stress (150 mM NaCl), and different concentrations of acetylcholine ; ACh (50 and 100 µM) under non-stress control and salinity-stressed conditions. The star mark; * indicates 5% level, ** indicates 1% level, *** indicates 0.1% level and **** indicates 0.01% level of significance, and ns indicates nonsignificant relationship between the studied parameters

Analysis of high affinity K^+^ transporter (HKT) encoding genes revealed significant up-regulation of *CaHKT1;1* and *CaHKT1;2* by salt stress (Fig. 8A and B). Exogenous ACh showed suppression of those under both non-stress control and salt stress conditions. As compared to non-stress control, expression of PS II- related transcripts like *CaPsbA*,* CaPsbB*,* CaPsbC and CaPsbD* were increased by many folds upon exogenous ACh under both control and salt stressed conditions (Fig. 8C and F). Salt stress itself also caused tremendous increment of those genes. However, the expression of *CaPsbD* was further boosted by ACh under salinity stress reflecting its crucial contribution under salinity stress (Fig. 8F). The expression of enzymatic antioxidant-related transcripts like *Cacu/ZnSOD*, *CaGR*, *CaAPX* and *CaCAT* were induced by salt stress and salt stress supplemented with ACh (Fig. 8G, H, J and K). However, as compared to only salt-stressed conditions, reduction of those transcripts’ expression upon ACh treatment reflecting the tremendous mitigation of salt-induced injury by ACh. The findings indicated the effective modulation of stress-related transcripts by ACh under both non-stress control and salinity- stressed conditions.

### Hierarchical heatmap analysis

**Fig. 9:**
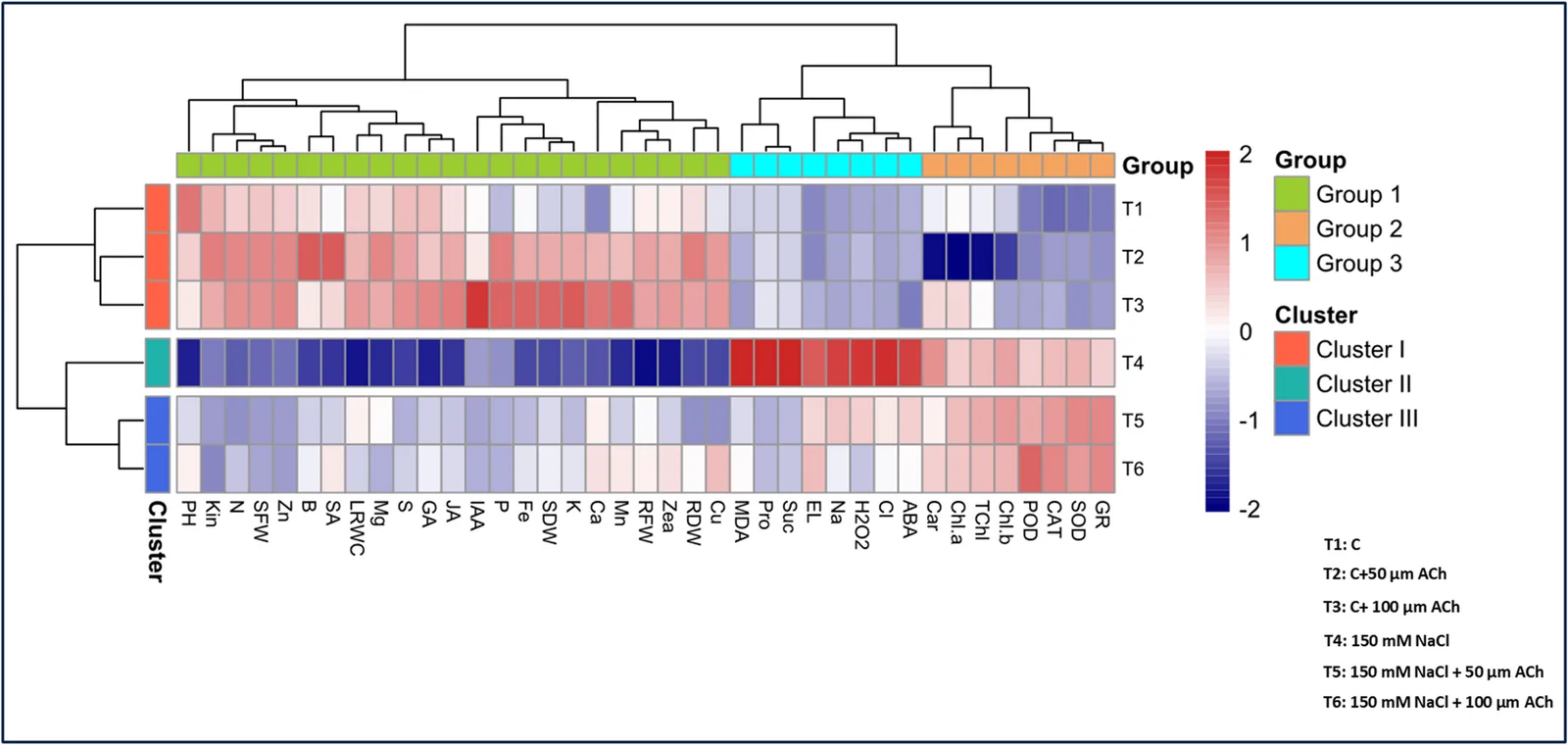
Hierarchical heatmap using morpho-physiological and biochemical parameters of pepper plant subjected to non-stress control (C), salinity stress (150 mM NaCl), and different concentrations of acetylcholine; ACh (50 and 100 µM) under non-stress control and salinity stressed conditions. Here, Mn; manganese, JA; jasmonic acid, SDW; shoot dry weight, Kin; Kinetin, RDW; root dry weight, SFW; shoot fresh weight, RFW; root fresh weight, IAA; indole-3 acetic acid, N; nitrogen, P; phosphorus, Fe; iron, Mg; magnesium, B; boron, Ca; calcium, K; potassium, Cl; chloride, GA; gibberellic acid, Zea; zeatin, S; sulfur, Zn; zinc, Na; sodium, Cu; cupper, PH; plant height, Car; carotenoids, Chl.a; *chlorophyll a*, Chl.b; *chlorophyll b*, Tchl; total chlorophyll, SA; salicylic acid, Pro; proline, ABA; abscisic acid, H_2_O_2_, Hydrogen peroxide, MDA; malondialdehyde, CAT; catalase, SOD; superoxide dismutase, Suc; sucrose, POD; peroxidase and GR; glutathione reductase

Hierarchical heatmap demonstrated three distinct groups of the studied parameters and three clusters of the treatment combinations applied in this study (Fig. 9). Group 1 included phenotypic parameters like PH, SFW, SDW, RFW, RDW, and nutrients elements like N, B, S, P, K, Ca, Mg, Cu, Zn and Fe, and phytohormones like Kin, SA, GA, JA, and IAA, and LRWC. The parameters of SOD, CAT, POD, GR, Chl *a*, Chl *b*, Car, and TChl were included in group 2, and EL, H_2_O_2_, MDA, Pro, Suc, Na, Cl, ABA were comprised with the group 3 (Fig. 9). Salinity stress (T4) producing negative regulation to the most of the studied parameters formed a different cluster (cluster II). Non-stress control (T1) and control with exogenous ACh (T2 and T3) having positive impacts to many studied parameters produced cluster I whereas salinity stress with different concentration of ACh (T5 and T6) having mitigating effects on plant phenotype, nutrients and antioxidant activities were supposed to cluster III (Fig. 9). 100 µM ACh showed to have best impacts on mitigating salt stress injury by ameliorating plant parameters, nutrient elements, phytohormone status, enzymatic antioxidants, and reducing ROS effects in terms of MDA, EL and H_2_O_2_ (Fig. 9). Details of the abbreviated forms were given in the figure caption (Fig. 9).

### Principal component analysis (PCA)

**Fig. 10:**
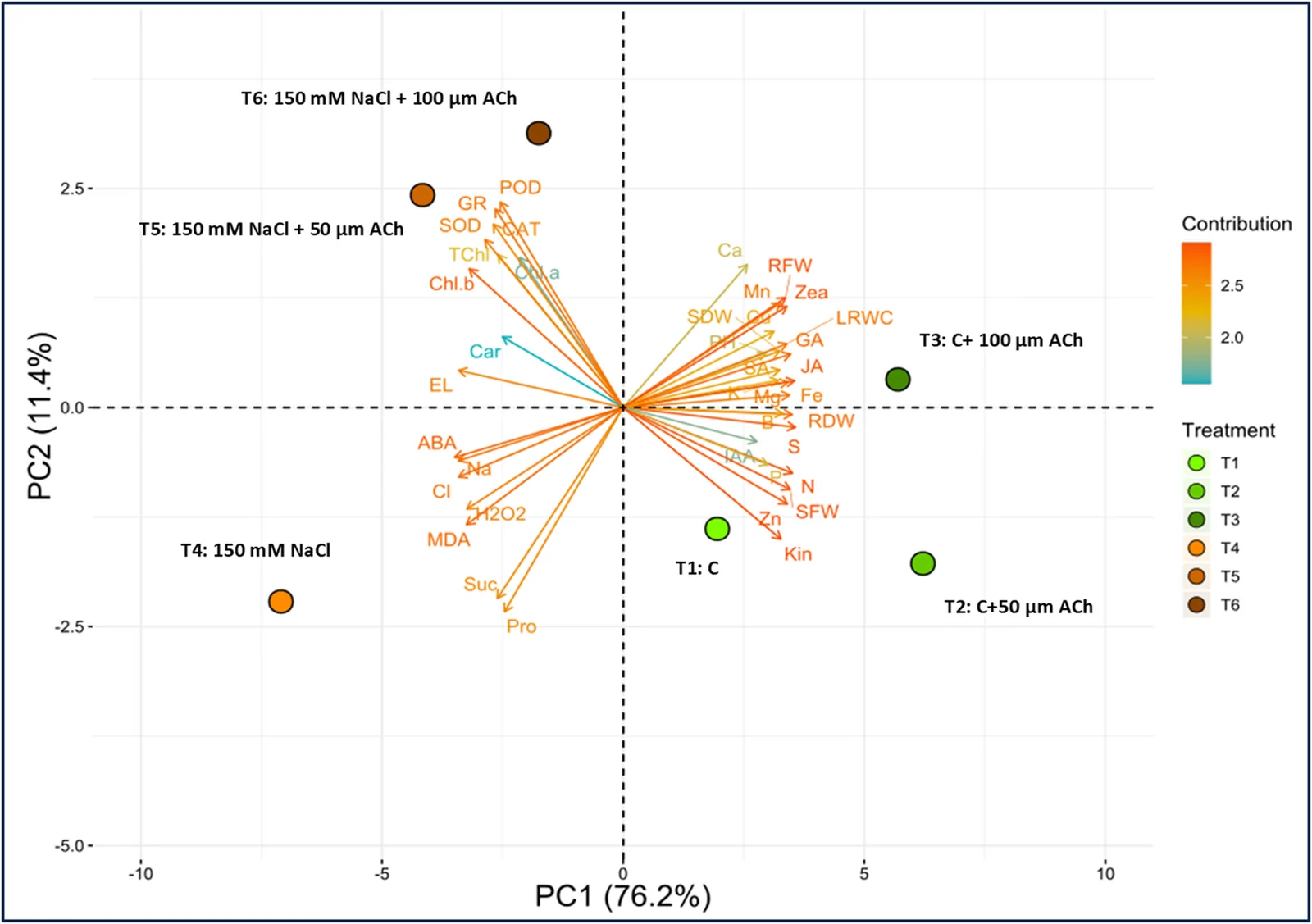
Principal component analysis (PCA) using morpho-physiological and biochemical parameters. The biplot of PCA stipulated relationship between studied parameters and treatment combinations. The contribution of the studied parameters to the principal component 1 (PC1) and principal component 2 (PC2) were demonstrated by the intensity of color and vectors’ length. The vectors’ angles formed from the center of biplot specified positive and negative interaction among them. Additional details were given in the caption of Fig. 9

The principal component analysis (PCA) of the studied parameters responding to different treatment combinations depicted that four principal components (PCs) of total five showed eigenvalues > 1, suggesting their valuable contribution in making variability of the parameters (Fig. 10 and Supp. Table 2). PC1 and PC2 showed 76.2% and 11.4% variability respectively, that combinedly produced 87.6% of the total variability (Fig. 10, Supp. Figure 1 and Supp. Table 2). In PC1, dependent variables like plant phenotypic characters, and LRWC, GA, JA, Fe, Mg, Zea, S, and N showed major positive contribution whereas, SOD, POD, CAT, Suc, Pro, ABA, H_2_O_2_, Na, and Cl showed negative contribution (Fig. 10). In contrast, LRWC, GA, JA, Fe, SOD, POD and CAT produced major positive contribution to PC2 where ABA, Na, Cl, H_2_O_2_, MDA, Suc, Pro, S, and Kin showed major negative contribution (Fig. 10). Salinity stress (T4) positioned in separate quadrate showed strong regulation on the variables like ABA, H_2_O_2,_ MDA Suc and Pro. Consistent to heat map, salinity stress with different concentration of ACh (T5 and T6) showed positive regulation on most of the biochemical parameters studied here (Fig. 10). Details of the abbreviated forms were given in the caption of Fig. 9.

## Discussions

Salinity stress has tremendous impacts on declining crop growth and productivity by disrupting various biochemical processes, including cell division, ionic balance, and synthesis of chlorophyll (Chl) molecules (Hao et al. 2021). The growth of the pepper plant was shown to decline under salinity stress (Yildirim et al. 2022; Ucar et al. 2026) which is consistent to this study where 100 mM NaCl sharply reduced plant phenotypes including shoot and root growth (Fig. 1). The limitation of water intake and ion toxicity might interfere the normal process of plant growth under salinity stress (Hassan et al. 2020; Tanveer et al. 2020). Stability of plant pigments like Chl and carotenoids (Car) are the vital indicators for growth and stress responses, where those pigments molecules are drastically disrupted by salt stress in many crops (Ashraf and Harris 2013; Dichala et al. 2021; Karimi et al. 2021). In contrast, the appearance of Chl and Car is somewhat fluctuating in the pepper plant during acclimation to salt stress. Although salinity at extreme level causes inevitably damage the structural integrity of photosynthetic pigments, many plants exhibit a resilient acclimation phase during low to moderate salt stress. Previous findings support that instead of an immediate decline, certain species actually stabilize or increase their chlorophyll levels to counteract the stress. For instance, total Chl content of mature leaves of *Cymbopogon nardus* was found to be considerably increased up to 200 mM NaCl but the level was further declined by increasing concentration (Mane et al. 2010). Accordingly, *Lavandula angustifolia* plant maintained enhanced level of Chl content when subjected to salt concentration below 200 mM, which is well corroborated to our findings where 150 mM NaCl showed relatively higher level of chlorophylls content compared to control plant (Fig. 2A and C). Such increase of Chl content in the leaves should be due to the retention of the osmotic adjustment capability of the plant by accumulating greater organic and inorganic compounds (Hmidi et al. 2018). However, it could also be happened by the greater ion compartmentalization ability of the plant (Peng et al. 2016). Alongside, Car having potential role in mitigating oxidative damage was greatly enhanced by salt stress (50 and 100 mM NaCl) in *Fagopyrum esculentum* (Lim et al. 2012; Li et al. 2025) which is well corroborated to us where 150 mM NaCl significantly induced Car (Fig. 2D). Unlikely, the level of Car in lavender plant was found to be decreased initially but increased with prolonged salt stress (Li et al. 2025), reflecting the potential role of this pigment molecule in photoprotection. ACh, a neurotransmitter, has been becoming as emerging weapon in plant systems by showing its positive responses in various plant processes including ion permeability (Leng et al. 2000), stomatal conductance (Wang et al. 2003), root initiation (Bamel et al. 2007), and growth enhancement (Murata et al. 2015). Along with growth, it had remarkable regulation in plant physiological and biochemical processes while acclimating to abiotic stresses (Qin et al. 2021). ACh ameliorated the negative consequences of salt stress, thereby rescuing plants from salt-induced damage (Su et al. 2020a; Qi et al. 2023; Shen et al. 2024). Likely, ACh-treated maize leaf showed increased relative growth rate in both control and arsenic-treated plants (Arikan-Abdulveli 2025). Here, ACh-induced restoration of pepper plant growth under both control and salt stress conditions showing a higher degree of compatibility with those findings. The enhanced growth could be due to the proper maintenance of more or less stable Chl *a* and Chl *b* and elevated level of TChl by ACh (Fig. 2). Upregulation of chlorophyll biosynthesis genes by ACh might play crucial role in this respect (Qin et al. 2020). However, modulation of growth responsive phytohormone auxin by neurotransmitter may trigger the activity of root meristem and overall plant growth (Amiri 2026).

Beyond damaging photosynthesis, high salinity triggers osmotic stress—one of the major constraints that lowers soil water potential. This potential drop makes it significantly harder for plants to absorb required water from the soil, leading to decreased relative water content (Parvin et al. 2019; Zhao et al. 2020). Maintaining leaf relative water content (LRWC) is crucial for plant resilience against abiotic stressors including salinity. LRWC (%) was shown to decline significantly in pepper plant while supposed to be salt-stressed condition (Aktas et al. 2012). This results mirrors to the present findings where LRWC (%) were significantly dropped down by 150 mM NaCl (Fig. 4A). However, plant accumulates compatible solutes like proline and soluble sugars to maintain osmotic balance and water retention capacity (Singh et al. 2022), that is closely aligned to this finding regarding enhanced levels of proline and sucrose by salinity stress (Fig. 4B and C). The same trend of osmolytes accumulation was demonstrated in pepper plant previously (Butt et al. 2016; Yildirim et al. 2022). Although ACh could potentially recover LRWC (%) in salt-affected plant but intriguingly it led to drastic drop down of proline and soluble sugar (Fig. 4). This indicates that ACh shifts the plant’s strategy from stress-defence to the growth of pepper plant by mitigating plant’s perception of osmotic stress (Ucar et al. 2026). Along with this study, dopamine and γ-aminobutyric acid (GABA)-led improved leaf water status under salt stress reflecting the crucial role of neurotransmitter in improving membrane integrity and ion permeability in plant (Yousaf et al. 2025; Ucar et al. 2026). However, improved hydration in leaf may also stem from the neurotransmitter-mediated nutrients availability and synergistic interaction with phytohormones (Qin et al. 2020; Qi et al. 2023).

The availability of macro- and micro-nutrients in plants are drastically hampered under salinity stress due to the prevalence of higher incidence of Na^+^ and Cl^−^. Higher levels of those potentially compete with essential nutrient elements like K, Ca, N, P, and Mg, leading to nutrients imbalance and deficiencies (Parihar et al. 2015). In agreement with Dere (2024), who noted that salinity stress cause higher incidence of Na^+^ and lower essential nutrient elements in pepper plant, this study found that excess NaCl cause drastic reduction of almost all the essential nutrient elements with elevated levels of Na^+^ and Cl^−^ (Fig. 3). Thus, the uptake of N and P could be reduced by the possible interaction of Cl^−^ raised by salinity. During salinity stress, an intense competition occurs between K^+^ and Na^+^, causing Na^+^ toxicity and leading to disrupting cell turgor, membrane integrity, and impairing K^+^ uptake that altogether is critical for metabolic processes and physiological adaptation of plants to abiotic stresses (Mostofa et al. 2022). Salt stress also caused a reduction of Ca which is vital for causing osmotic balance, membrane stability, and stress adaptation processes (Park et al. 2016). Exogenous application of neurotransmitter like ACh and dopamine strongly help maintain lower Na^+^/K^+^ ratio by declining Na⁺ availability in pepper plant (Qin et al. 2020; Ucar et al. 2026). Consistently, higher level of K⁺/Na⁺ in this study by ACh reflecting the potential role of neurotransmitter in diminishing salinity effects in pepper plant. Given that, higher level of cytosolic K⁺/Na⁺ could boost a plant’s ability to minimize Na⁺ toxicity and withstand salinity stress (Park et al. 2016; Wu 2018). Alongside, ACh potentially enhanced the level of Ca, Mg, S, Mn, Zn, B, Fe and Cu in pepper plant under salinity stress (Fig. 3) which could have tremendous impacts on preserving LRWC, osmotic balance and overall plant growth and development (Figs. 1, 3 and 4). This could be managed by the lowering of Na^+^ toxicity in pepper plant by ACh. However, tremendous improvement of plant nutrient status by ACh in non-stressed pepper plant (Fig. 3), suggesting its potential contribution in plant growth and development, although the exact mechanism of that needs to be clarified further.

Along with disrupting nutrient status, salinity stress promotes the generation of ROS, particularly H_2_O_2_, causing damage to plant tissues (Qin et al. 2020). Accordingly, maize seedling produced elevated levels of foliar H_2_O_2_ under salt stress which is aligned to this finding showing elevated level of H_2_O_2_ in pepper leaf subjected to salt stress (Fig. 5A). Enhanced level of H_2_O_2_ induced by abiotic stresses causes tissue damage even cell death by disrupting membrane integrity and triggering lipid peroxidation; MDA (Ghosh et al. 2021). However, increased level of MDA caused profound membrane damage leading to the greater electrolyte leakage (EL) (El-Esawi et al. 2018). Significantly higher levels of MDA and EL (%) in pepper leaf in this study reflecting the potential level of ROS-induced injury incurred by excess NaCl. Such disruption by ROS likely stems from an ionic imbalance (higher Cl⁻ and lower K^+^) within the cytosol, which potentially disrupts optimal chloroplast functioning (Hedrich and Shabala 2018). Application of ACh could dramatically reduce the incidence of ROS and thereby reduced level of MDA and EL (%) in pepper leaf (Fig. 5), which was supported by the findings of Qin et al. (2021) who demonstrated that occurrence of H_2_O_2_ and O_2_^•−^ were markedly reduced by ACh in the leaves of salt-stressed plants. Concomitantly, Shen et al. (2024) documented the reduction of ROS incidence in maize leaf by exogenous ACh, which altogether suggesting the potential role of ACh in mitigating salt-induced ROS in plant. Multivariate analyses using biochemical parameters further supported the potential of exogenous ACh in destabilizing ROS in pepper plant (Figs. 9 and 10). This could be happened by accelerating activities of enzymatic antioxidants by exogenous neurotransmitter in salt- affected plants (Liu et al. 2025; Qin et al. 2019). Intriguingly, salt stress itself led to the enhancement of antioxidant activities in pepper plant in terms of elevated levels of SOD, CAT, POD and GR (Fig. 6) but these were not sufficient to fight against boosted ROS as provoked by notorious salinity stress (Fig. 5). Those enzymatic antioxidants are vital for establishing antioxidant defence in plant, because SOD makes first line defence by dismutating O_2_^•−^ into H_2_O_2_ (Che et al. 2020), while H_2_O_2_ turns to H_2_O with the help of CAT and APX (Cao et al. 2023). Supplementation of neurotransmitters like ACh, dopamine and GABA could potentially lower H_2_O_2_ and MDA by triggering antioxidant system in salt-affected plants (Qin et al. 2019; Alnusaire, 2026; Ucar et al. 2026) that potentially mirrors to this finding where exogenous ACh significantly enhanced the activities of SOD, CAT, POD and GR in salt-stressed pepper plant. Along with salinity stress, exogenous ACh was demonstrated to boosts up antioxidant enzymes while counter facing other abiotic stresses including drought and metal stress (Qi et al. 2023; Saleh 2024), suggesting its potential role in ROS homeostasis under a wide range of abiotic stresses. Concurrently, enhanced activities of APX and GR in pepper’s leaf by ACh engaging AsA-GSH cycle enzymes with this defence system (Fig. 6). Promotion of GR might ensure continuous supply of glutathione (GSH) which acts as antioxidant directly and potentially serves as the substrate for APX, indeed crucial for operating AsA-GSH cycle (Arikan-Abdulveli 2025).

Along with that, phytohormones like Auxin, cytokinin, GA, JA and SA are crucial to address salinity tolerance in plants (Yu et al. 2020). Salinity stress showed drastic reduction of essential phytohormones like IAA, GA, Kin, Zea, JA and SA (Fig. 7A and F) reflecting its tremendous impacts on impairing plant’s metabolic process, and growth and development. Accelerated level of GA by ACh could support plant morpho-physiological attributes resulting to enhanced plant growth and salinity tolerance (Shahzad et al. 2021). Likely, ACh-induced IAA in pepper optimized plant growth under non-stress control and improved root growth plasticity in response to salinity stress (Liu et al. 2015). Concomitantly, higher level of cytokinins particularly Zea by ACh in salt-stressed pepper plant likely to be associated with cytokinin-mediated shoot Cl^−^ exclusion (Yin et al. 2023), and improvement of antioxidant activities (Azzam et al. 2022). However, salinity-exposed pepper plant improved JA synthesis which may increase tolerance by improving K^+^ and Ca^2+^ and declining Na^+^ (Taheri et al. 2020). In this line of research, ACh elevated SA in plant that could improve salinity tolerance by showing positive regulation to AsA-GSH cycle enzymes (Figs. 6 and 7). Notably, ACh showed sharp increment of phytohormones except ABA in non-stress control plant indicating its potential role for improving pepper plant growth. Conversely, salinity significantly elevated the level of ABA (Fig. 7G), which exerts an extensive regulatory role over physiological mechanisms of stress adaptation (Takezawa et al. 2015). Indeed, this increased level of ABA assists salt-stressed plant by triggering antioxidant enzymes like SOD, CAT, POD and APX (Chen et al. 2022). Intriguingly, the synthesis of ABA was further declined by ACh under salt stress indicating the lowering of stress-effects by this phytochemical. This decline could also be happened by the antagonisms of ABA with GA (Qin et al. 2021) and cytokinins (Pospíšilová 2003) as raised by ACh under salinity (Fig. 7). Thus, the present findings reinforced the regulatory role of ACh on engaging phytohormones to combat salinity stress and restoring plant growth. Despite having crosstalk between phytohormones and ACh in regulating plant growth and stress acclimation process (Qin et al. 2021; Shen et al. 2024), the mechanistic insight how ACh regulate phytohormone synthesis remains to be explored. Transcriptomics and proteomics analyses using ACh transgenics and mutants under salinity stress might explore that mechanisms.

When a plant gets salinity stress, it employs high-affinity potassium transporters (HKTs) that strongly regulate K^+^ and Na^+^ transport in plants. For instance, class I *HKT1;1* was reported to assist Na^+^ exclusion from the shoot by retrieving and transporting Na^+^ back towards root xylem (Ali et al. 2021; Gharaghanipor et al. 2022). In monocot species, some members of the HKT2 subfamily have been associated with Na^+^ transport and distribution under salinity stress (Mian et al. 2011). Therefore, HKT transporters have been established as the crucial regulators of salinity tolerance in both monocotyledonous and dicotyledonous plant species, including rice (Kobayashi et al. 2017), wheat (Byrt et al. 2014), maize (Zhao et al. 2023), barley (Hazzouri et al. 2018), tomato (Romero-Aranda et al. 2020), strawberry (Garriga et al. 2017), lettuce (Santander et al. 2021), and poplar (Xu et al. 2018). In this study, salt-stressed pepper plants showed a significant induction of class I *HKT1;1* and *HKT1;2* compared with non-stressed control plants (Fig. 8A and B), suggesting activation of HKT-mediated ion transport and Na^+^ homeostasis mechanisms under saline conditions (Hauser and Horie 2010). Jaime-Pérez et al. (2017) reported that *HKT1;2* is expressed in the vascular system, including xylem and phloem cells of tomato leaves and roots, while *HKT1;1* is expressed only in the leaves. Exogenous ACh has been shown to influence plant growth and development by regulating the expression of stress-responsive genes (Di Sansebastiano et al. 2014). Although ACh was previously reported with the regulation of ion homeostasis-related pathways under salinity stress (Qin et al. 2019), the reduced expression of *CaHKT1;1* and *CaHKT1;2* following ACh treatment may reflect alleviation of ionic toxicity and diminished requirement for stress-induced HKT1 activation in pepper plants. The fact was supported by the dopamine-mediated tremendous downregulation of HKT transcripts in salt-affected pepper’s leaf (Ucar et al. 2026). Therefore, neurotransmitters might help maintain Na^+^/K^+^ balance by modulating HKT-associated ion transport pathways under salinity stress.

Alongside, transcripts encoding PS II core complex proteins like CP47 (PsbB) and CP43 (PsbC), and D1 (PsbA) and D2 (PsbD) subunits of PS II reaction centre were found to be greatly enhanced by both salinity stress and ACh (Fig. 8C and F) reflecting the possible interaction of salinity stress and ACh while repairing photosystem. The findings was supported by the enhanced expression of *LaPSBC* (encoding PS II CP43 reaction centre protein), *LaPSBY-1*,*2* (encoding PS II core complex protein), and *LaPSB28* (encoding PS II reaction centre protein) by salinity stress (Zhao et al. 2024; Li et al. 2025) suggesting that those genes are essential to combat salinity- induced damage to photosystem. Notably, tremendous upregulation of *CaPsbD* by ACh in salt-stressed pepper plant (Fig. 8F) suggesting stabilizing PS II functioning by this phytochemical. Besides, plants are experienced with higher level of antioxidant enzyme- related transcripts such as *OsSOD3Cu/Zn*, *OsSODA1-Mn*,* OsSOD4-Cu/Zn*, *OsSOD3-Cu/Zn*, *OsSODCc1-Cu/Zn*, *OsSOD-Fe*, *OsSODA1-Mn*, *OsCATA*, *OsAPX2* and *OsGR1* to counteract salinity stress (Vighi et al. 2017). Aligned to these results, this finding showed many folds increment of *CaCu/SnSOD*, *CaGR*, *CaAPX* and *CaCAT* by both salinity and ACh in pepper plant (Fig. 8G, H and J, and 8K). However, the declined trend of *CaCu/SnSOD*, *CaGR* and *CaCAT* expression by ACh under salt stress could be due to higher expression of other isoforms which may compensate the elevated level of antioxidant enzymes (Fig. 6). ACh-mediated tremendous increment of HKT transporter, PS II- and antioxidant- related transcripts in non-stress control plant suggesting strong modulation of those genes by this phytochemical in pepper plant. Alongside, our multivariate analysis through hierarchical heat map clearly depicted that both 50 and 100 µM ACh showed positive impacts on mitigating salinity stress where 100 µM ACh showed better positive regulation by improving most of the physiological parameters particularly essential nutrients content, phytohormones and antioxidant enzymes, and destabilizing excess ROS incurred by salinity (Fig. 9). The statement was supported by the results of PCA where exogenous ACh (100 µM) produced most stunning effects in regulating physiological and biochemical parameters studied here (Fig. 10). Thus, the present findings imply that exogenous ACh (100 µM) is crucial for plant growth, development and mitigating salt stress in pepper plant. Further field trial with a broad ecological application is needed to validate this eco-friendly chemical technology for improving productivity under salinity stress.

## Conclusion

Salinity stress severely restricted growth and development of pepper plant by attenuating LRWC, essential nutrients, and phytohormones content while inducing ROS-led damage. Salinity promoted accumulation of osmolytes, activities of enzymatic antioxidants and expression of transcripts regarding K^+^ transporters, PS II and enzymatic antioxidants. Exogenous ACh profoundly improved LRWC, nutrient status, and phytohormones content in both control and salinity stress conditions. It could maintain redox balance by enhancing the activities of SOD, CAT, POD, and GR, and destabilizing excess ROS under salinity stress. However, both salinity and ACh showed strong modulation on the expression of transcripts related to K^+^ transporters, PS II and antioxidant enzymes. Thus, ACh, particularly at 100 µM, is crucial for improving plant growth under non-stress conditions and for physiological adaptation to salinity stress. Finally, the findings underscore a promising strategy for the sustainable growth of pepper plant and strengthening future food security. Nevertheless, future efforts employing multi-omics analyses of ACh-related transgenics under salinity stress are required to elucidate the precise molecular pathways involving ACh-mediated stress response in plant. 

## Supplementary Information

Below is the link to the electronic supplementary material.

Supplementary Figure 1. Scree plot presenting percentage of explained variances produced by the different dimensions of Principal component analysis (PCA) (PNG 36.1 KB)

High Resolution Image (TIF 195 KB)

Supplementary Material 2 (DOCX 23.7 KB)

Supplementary Material 3 (DOCX 20.5 KB)

## Data Availability

The datasets generated and/or analyzed during the current study are available from the corresponding author on reasonable request.
