# Supplementary material for: Acetylcholine as a shield: enhancing growth and salt tolerance in pepper through ionic homeostasis, hormonal regulation, antioxidant defence and gene expression
Source: Protoplasma. 2026 May 28;263(5):1711–28. doi: 10.1007/s00709-026-02223-9 (PMC13493410; doi:10.1007/s00709-026-02223-9)
Supplement: Supplementary file 1 [file 709_2026_2223_MOESM2_ESM.docx]

**Supplementary Table 1.** List of primers’ sequences used for qPCR assay.

| **Gene** | **Forward Primer** | **Reverse Primer** | **Reference** |
| --- | --- | --- | --- |
| *CaHKT1;1* | GGCATTCATATCAGTTCAGTTTGT | TTATCAACAGGCAAAAAAGTAGTAGAG | [Zhang et al. (2020)](https://www.sciencedirect.com/science/article/pii/S0981942825003262?casa_token=tJvPcqXQ-nMAAAAA:JN0KE3qIvlzCTEv8D4SQIvuTX5bxLuUaFEvf1aBNYwvNGQmo3-PR4KOgFWP_UexZi1LaQ0qHAfFx#bib105) |
| *CaHKT1;2* | AAACACAGTGTATTGCAGAACAACGAT | TCAAGATTATGAAGACCTTCACCATTA |  |
| *CaPsbA* | GAATAGGGAGCCGCCGAATACAC | TATTCCAGGCTGAGCACAACATCC | [Tang et al. (2021)](https://www.sciencedirect.com/science/article/pii/S0981942825003262?casa_token=tJvPcqXQ-nMAAAAA:JN0KE3qIvlzCTEv8D4SQIvuTX5bxLuUaFEvf1aBNYwvNGQmo3-PR4KOgFWP_UexZi1LaQ0qHAfFx#bib89) |
| *CaPsbB* | TGGGTTTGCCTTGGTATCGTGTTC | GCCCAACCAGCAACCAGAGC |  |
| *CaPsbC* | GGATCTGCGTGCTCCATGGTTAG | CCGTTCCTGCCAAGGTTGTATGTC |  |
| *CaPsbD* | TGGTCACCGCTAACCGCTTTTG | AGACCGACTACTCCAAGAGCACTC |  |
| *CaCu/ZnSOD* | TGTCGCCGTCCTTAGCAGC | TTCCGCCAGCGTTTCCAGT | Deng et al. (2012a) |
| *CaPOD* | ATGCCCTGGTGTTGTTTC | CCTTGGCTTGCTGTCCTG |  |
| *CaAPX* | TCCTATTATGCTCCGTCTCG | AACAGGTGGTTCTGGCTTG | Deng et al. (2012b) |
| *CaGR* | ATGGATGGGAAGTGAATGAGA | AGAATGTGCTTTGCCGAATAG | Wang et al. (2016) |
| *CaCAT* | GTTGGTCGCTTGGTATTGA | TTGGGAGCGTTAGGTGG | Ali et al. (2020) |
