# Supplementary material for: Acetylcholine as a shield: enhancing growth and salt tolerance in pepper through ionic homeostasis, hormonal regulation, antioxidant defence and gene expression
Source: Protoplasma. 2026 May 28;263(5):1711–28. doi: 10.1007/s00709-026-02223-9 (PMC13493410; doi:10.1007/s00709-026-02223-9)
Supplement: Supplementary file 2 [file 709_2026_2223_MOESM3_ESM.docx]

**Supplementary Table 2:** Various dimensions, eigen values, variances (%) and cumulative variance (%) of the principal component analysis (PCA) using morpho-physiological and biochemical parameters analyzed in this study

| **Dimensions or prinipal components** | **Eigen values** | **Variance (%)** | **Cumulative variance (%)** |
| --- | --- | --- | --- |
| Dim.1 | 29.7 | 76.2 | 76.2 |
| Dim.2 | 4.5 | 11.4 | 87.6 |
| Dim.3 | 2.3 | 5.9 | 93.5 |
| Dim.4 | 2.0 | 5.0 | 98.5 |
| Dim.5 | 0.6 | 1.5 | 100.0 |
